# Intracranial choriocarcinoma occurrence in males: Two cases and a review of the literature

**DOI:** 10.3892/ol.2013.1570

**Published:** 2013-09-09

**Authors:** JIANYI GUO, CHUNLONG ZHONG, QIANG LIU, JIWEN XU, YAN ZHENG, SIYI XU, YANG GAO, YANG GUO, YONG WANG, QIZHONG LUO, JIYAO JIANG

**Affiliations:** 1 Department of Neurosurgery, Renji Hospital, Shanghai Jiao Tong University School of Medicine, Shanghai 200127, P.R. China; 2 Department of Pathology, Renji Hospital, Shanghai Jiao Tong University School of Medicine, Shanghai 200127, P.R. China

**Keywords:** intracranial choriocarcinomas, male, tumorigenesis, prognosis

## Abstract

Choriocarcinomas generally develop in females. Non-gestational choriocarcinoma in males is extremely rare. The present study describes two cases of young males who were diagnosed with intracranial choriocarcinoma. One case was of an aggressive choriocarcinoma with multiple metastases to the brain, but with an unidentified origin. The patient was admitted in the terminal stage of the cancer. Although a tumor resection was performed, the condition of the patient rapidly deteriorated and chemotherapy was not recommended. The patient succumbed nine days after the surgery. The second case was of a primary ventricular choriocarcinoma. The patient was hospitalized for acute hydrocephalus caused by a mass that was located in the ventricle. Following a tumor resection, the patient underwent a course of whole-brain and spinal radiotherapy. The patient was followed up for more than half a year and remained in a good condition. The present study describes the two cases and a comprehensive review of the literature that was performed to identify similar studies that document choriocarcinomas in males.

## Introduction

A choriocarcinoma is a highly malignant tumor arising from the embryonal chorion. Generally, the term refers to gestational choriocarcinoma, which most commonly occurs with a hydatidiform mole, spontaneous abortion, ectopic pregnancy or normal delivery (1). Choriocarcinoma is a cancer that typically occurs in females in the chorionic epithelium of the placenta (2) and is rarely seen in males. The Department of Neurosurgery, Renji Hospital (Shanghai, China) treated two male patients with non-gestational choriocarcinoma between 2003 and 2008. One patient was diagnosed with disseminated brain metastases with an unknown origin and the other patient was diagnosed with a primary intracranial third ventricle choriocarcinoma. Written informed consent was obtained from the patients.

## Case reports

### Case 1

**Figure 1:**
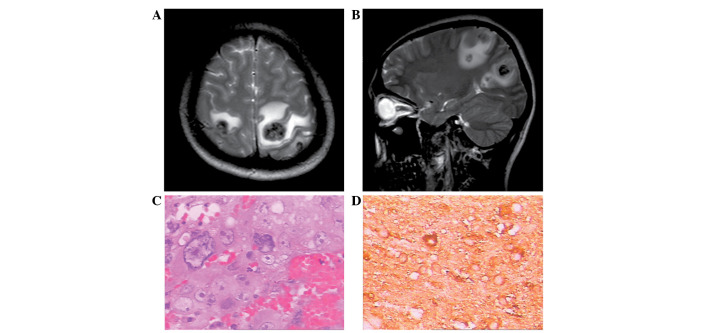
Case 1. Pre-operative (A) axial and (B) sagittal magnetic resonance imaging (MRI). Multiple lesions were observed in the bilateral parietal and occipital lobes. (C) Photomicrograph showing cytotrophoblastic cells and multinucleated syncytiotrophoblastic cells characteristic of choriocarcinoma (HE staining; magnification, ×400). (D) Syncytiotrophoblastic cells indicating a positive reaction for β-human chorionic gonadotropin (β-hCG).

A 21-year-old male with a two-month history of dizziness and fever and a one-week history of vomiting and numbness of the right limbs was admitted to the Renji Hospital (Shanghai Jiao Tong University School of Medicine, Shanghai, China). The patient developed symptoms of dizziness and fever two months prior to being admitted. At that time, the patient was treated in the local clinic with a course of anti-inflammatory medication. The symptoms improved, but a mild fever remained. The chest X-ray indicated nothing unusual at that time. One week prior to admission, the patient complained of coughing and sputum with occasional blood streaks, left upper limb pain and numbness of the right limbs. The patient also vomited frequently. The vomitus contained gastric contents. A chest X-ray was performed again at the outpatient department and revealed multiple nodules in the lungs. The brain magnetic resonance imaging (MRI) scans demonstrated multiple mass lesions occupying the bilateral parietal and occipital lobes (Fig. 1A and B). The past history was unremarkable. Upon physical examination, the patient was identified to be normotensive with a temperature of 37.8°C. There were no abnormal signs on the nervous system examination. The superficial lymph nodes were not palpable. There were no abnormal findings upon physical examination of the thorax and abdomen. A further abdominal ultrasound detected hyperechoic masses in the liver and kidneys.

Intracranial surgery for the resection of the multiple lesions was performed one day after admission. The tumor appeared dark red with a tough texture and rich blood supply. The boundary with the normal brain tissue was clear. An intra-operative biopsy of the mass was sent for frozen section analysis and the result was that of a metastatic poorly-differentiated cancer. Following the surgery, the patient gradually developed intracranial hypertension symptoms, including headaches and projectile vomiting. The symptoms progressively worsened. On post-operative day seven, a head computed tomography (CT) scan indicated that new hemorrhagic metastases had emerged in the frontal and parietal lobes. The parents of the patient rejected a decompressive craniectomy and chose a conservative treatment. The pathology report revealed that the brain tumor was a metastatic choriocarcinoma and the immunohistochemistry is shown in Fig. 1C and D. The urine was strongly positive for β-human chorionic gonadotropin (β-hCG) and the serum β-hCG was markedly elevated at 16,500 mIU/ml. The testicles were equal in size and ultrasonography did not reveal anything abnormal. Following a consultation with the Department of Oncology, conservative therapy was suggested, since the patient was too ill for chemotherapy. On the day of the consultation, the situation deteriorated with unstable vital signs. Two days later, the patient succumbed to the disease.

### Case 2

A 20-year-old male was admitted to the Renji Hospital due to a sudden onset of unconsciousness, with a six-month history of nausea and vomiting. The neurological findings were unremarkable, with the exception of a low level of consciousness. A head CT scan demonstrated acute hydrocephalus and a mass lesion occupying the third ventricle.

**Figure 2:**
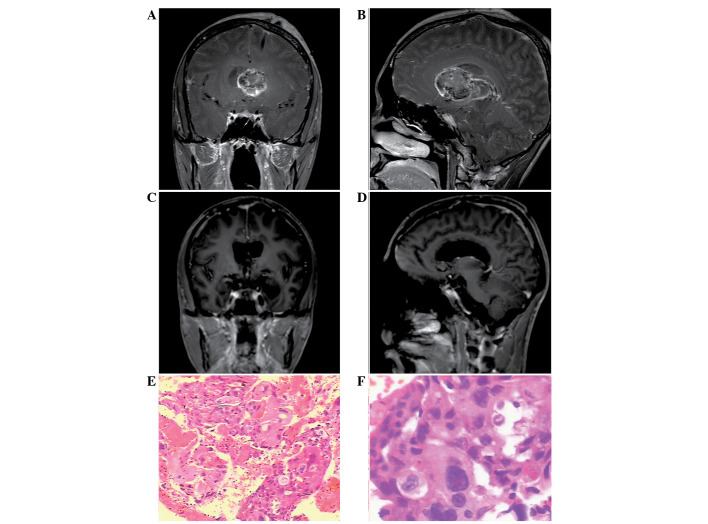
Case 2. Pre-operative (A) coronal and (B) sagittal enhanced magnetic resonance imaging (MRI). An irregularly-enhanced large mass lesion was observed in the third ventricle. (C) Coronal and (D) sagittal MRI obtained following radiotherapy. The majority of the tumor had been resected. (E and F) Photomicrographs showing cytotrophoblastic cells and multinucleated syncytiotrophoblastic cells characteristic of choriocarcinoma (HE staining; magnification, ×100 and ×400, respectively).

Emergency surgery for external ventricular drainage was performed upon admission. Following the surgery, the consciousness level improved. A head MRI examination was then performed and revealed a mass lesion in the ventricle (Fig. 2A and B). The chest X-ray and abdominal ultrasound revealed nothing unusual. A mass resection surgery was performed. The histological diagnosis of the surgical specimen was that of a choriocarcinoma (Fig. 2E and F). The ultrasound of the testicles did not reveal anything abnormal. The serum β-hCG concentration was 278 mIU/ml and the urine was weakly positive for β-hCG. One month later, the serum β-hCG level had risen to 2,760 mIU/ml. Whole-brain and spinal radiation was initiated when the patient was in a stable condition. Over 36 days, the patient was administered a cumulative dose of 36 Gy whole-brain irradiation and a 14-Gy local boost. The total dose was 50 Gy/27 Fx/36 days. An MRI scan demonstrated that the tumor mass had shrunk during the course of the radiotherapy (Fig. 2C and D). The patient was followed up for more than half a year and remained in a good condition.

## Discussion

Choriocarcinomas are aggressive malignancies that are grouped into two categories, gestational choriocarcinoma, which is derived from any form of previously normal or abnormal pregnancy, including a hydatidiform mole, spontaneous abortion or ectopic pregnancy, and non-gestational choriocarcinoma, which typically arises from gonadal organs, but also occurs rarely in extragonadal primary sites. The present study described two cases of non-gestational choriocarcinoma. One case was of an aggressive choriocarcinoma with multiple metastases to the brain, but with an unidentified origin, and the other case was of a primary intracranial choriocarcinoma.

Non-gestational choriocarcinoma typically arises from the gonadal organs. When the primary origin is extragonadal, choriocarcinoma occurs preferentially at the midline section, including the pineal body, mediastinum and retroperitoneum (3). In the rare occurrences in males, choriocarcinoma is most commonly observed in the testes. A total of 106 male choriocarcinoma cases were reported between 1995 and 2006 (4). The testis was the most common primary site in 33.0% (35/106). Metastasis was common, with a frequency of 83% (81/98, the data from eight patients were missing). The majority of the cases included multiple metastases. The most common metastatic sites were the lung, liver and brain, and the metastases progressed rapidly.

Several theories explaining the pathogenesis of these extragonadal choriocarcinomas have been proposed, but no conclusions have yet been reached. There are presently three hypotheses: i) The tumor is a metastasis from a testicular choriocarcinoma that regressed spontaneously (5); ii) the tumors arise from the primordial germ cells that migrate abnormally during embryonic development, which may explain the choriocarcinomas that originate from organs that are remote from the genital tract (6); and iii) the tumor is a cancer that develops originally as a non-trophoblastic neoplasm and is transformed into a choriocarcinoma (7).

A diagnosis is possible if the site is near the body surface, and a partial or total biopsy may be performed relatively safely. However, a biopsy may not be performed at a number of sites, including the mediastinum, pineal body, lung and retroperitoneum, making a pre-operative diagnosis extremely difficult. When a diagnosis of non-gestational choriocarcinoma is suspected, it is necessary to fully examine the patient’s testes. Generally, the physical examination of testicular tumors includes palpation for testis enlargement and occasionally, an assessment of trigger points for pain.

Furthermore, since non-gestational choriocarcinoma has a trophoblastic element, the tumor secretes β-hCG. Therefore, the tumor is associated with a markedly raised serum β-hCG concentration, which is significant in the diagnosis and monitoring of the clinical progress. Yokoi *et al*(4) reported that 96.4% of choriocarcinoma patients have abnormally elevated serum β-hCG. This percentage indicates that the test is highly precise. In the patient from case 1, the urine β-hCG was strongly positive and the serum β-hCG concentration was 16,500 mIU/ml. This is consistent with the aforementioned feature of choriocarcinoma. The serum β-hCG concentration in the patient from case 2 was 278 mIU/ml, and one month later, the concentration rose to 2,760 mIU/ml. An increased level was not as evident as with the patient of case 1. This finding may be explained by the resection of the primary site. In addition, since the assay for β-hCG concentration is easy to perform and non-invasive, it may be repeated and performed quickly, placing little burden on the patient.

The clinical manifestation of non-gestational choriocarcinoma is varied. The cancer from the primary site spreads via the blood and lymphatics, with early hematogenous dissemination to the lungs, liver, brain and other visceral sites (8). The usual mode of presentation in a testicular choriocarcinoma is testicular enlargement, occasionally with pain. Common metastatic symptoms include hemoptysis secondary to pulmonary metastases, back pain secondary to retroperitoneal spread, gastrointestinal bleeding due to gastrointestinal tract metastases and neurological symptoms from brain metastases (9).

It is evident that an early diagnosis was not established in the first case, and the condition of the patient deteriorated prior to the appropriate therapy being instituted. Subsequent to the pathology results confirming the diagnosis, a physical examination and ultrasound of the testis was performed, and nothing abnormal was identified. Since an autopsy was not allowed, the primary site was not identified. According to the ‘burned out tumor’ theory, as the primary site in the testis may be quite small or even totally regressed, a clinical examination of the testis may appear normal (10). Therefore, the presumed primary site was the testis. Furthermore, the second patient was diagnosed at a relatively early stage.

A total or subtotal resection of an intracranial choriocarcinoma is useful for the prognosis of the patient. Advances in neuroimaging, microsurgical techniques, surgical instruments and surgery-supporting systems allow a subtotal or greater resection of an intracranial choriocarcinoma to be feasible and safe. However, the total removal of the tumor remains difficult. Radiotherapy is an indispensable therapeutic modality for intracranial choriocarcinoma. Kohyama *et al*(11) reported a successfully treated case that underwent stereotactic radiation therapy. Stereotactic radiation therapy was undertaken for the residual tumor of the pineal region following a partial tumor removal, external radiotherapy and chemotherapy, and the patient has been in a good condition for more than four years. An increased risk of impaired brain function due to radiotherapy has been recognized in young patients with brain tumors, and the minimum dose of irradiation supplemented with chemotherapy is now recommended (12). In Japan, a phase II study of combined chemotherapy and radiotherapy was undertaken for choriocarcinoma (14 neurosurgical clinics in Japan). In the study, following a surgical tumor resection or subtotal resection, a combination chemotherapy using carboplatin, etoposide and ifosfamide followed by whole brain and spinal radiotherapy with doses of 30 Gy and a 30 Gy boost delivered to a generous local field was used. Thereafter, additional chemotherapy with the same regimen was repeated a total of five times every three to four months (13).

Choriocarcinoma is one of the most aggressive and malignant germ cell tumors and the clinical outcome is poor. An investigation based on 97 cases revealed that the mean survival time was 7.7 months and that the one-month mortality rate was 23.8% (4). As the patient of case 1 illustrated, the cancer had already metastasized to multiple organs, including the brain, lungs, liver and kidneys, when the patient was admitted, and the choriocarcinoma was identified at the terminal stage. At post-operative day 7, the head CT scan demonstrated new metastatic lesions that accompanied hemorrhaging. The symptom of intracranial hypertension was grave and the patient succumbed on post-operative day 9.
